# A comprehensive review of lateral flow assays in rapid livestock and poultry disease diagnostics

**DOI:** 10.5455/javar.2026.m1032

**Published:** 2026-03-24

**Authors:** Sajedul Hayat, Md. Enamul Haque, Mohammad Sadekuzzaman, Susmita Karmakar, Md. Ariful Islam, Muhammud Tofazzal Hossain, Md. Alimul Islam

**Affiliations:** 1 Department of Microbiology and Hygiene, Bangladesh Agricultural University, Mymensingh, Bangladesh; 2 Department of Biochemistry and Molecular Biology, Dr. Momtaz Begum University of Science and Technology, Bhairab, Kishoreganj, Bangladesh; 3 Central Disease Investigation Laboratory, Department of Livestock Services, Dhaka, Bangladesh

**Keywords:** User-friendly identification, livestock, poultry, animal, zoonotic diseases, sustainable

## Abstract

Lateral flow assays (LFAs) are a vital tool for rapid, simple, and low-cost disease detection in poultry and livestock. LFAs became a widely used approach in veterinary diagnostics for better animal health management. In this study, we thoroughly highlight the concepts, recent developments, and applications of LFAs in the expedited diagnosis of livestock and poultry diseases. LFAs are portable, require minimal sample preparation, and can be used for point-of-care testing, making them ideal for resource-limited field settings. In this article, we examined various types of LFAs, including multiplex platforms, nucleic acid assays, and antigen-antibody-based detection approaches. We also emphasized their significance in identifying infections that threaten animal welfare, caused by viruses, bacteria, and parasites. Recent advancements in LFA have significantly improved sensitivity and specificity, incorporating quantitative analysis, smartphone-compatible readout devices, and nanomaterials. This paper also addresses problems with false positives, the limited number of multiplexing situations it can handle at a time, and the need for enhanced stability across a wide range of environmental conditions. Furthermore, they investigated the possibility of LFAs satisfying the growing need for rapid disease diagnosis and surveillance, particularly in light of emerging zoonotic diseases. This review emphasizes the vital role of LFAs in improving diagnostics for livestock and poultry diseases by summarizing current trends and identifying research needs, thereby supporting global animal health and sustainable agricultural practices.

## 1. Introduction

Countries with largely agricultural-based economies face significant challenges from transboundary and zoonotic diseases, which affect not only agriculture but also livestock and public health [1]. Endemic transboundary diseases such as foot-and-mouth disease (FMD), avian influenza A (AIV), hemorrhagic septicemia (HS), peste des petits ruminants (PPR), and lumpy skin disease (LSD) pose a significant challenge in the agricultural sector, resulting in substantial economic losses [2]. Moreover, 60% of human infections are considered zoonotic, and 75% of emerging human diseases detected in the past three decades are of animal origin [3]. Both bacterial and viral zoonoses pose an imminent threat to the nation. Among viral zoonotic diseases, avian influenza, rabies, Nipah virus infection, Japanese encephalitis, rotavirus, and dengue fever are particularly prevalent. At the same time, bacterial zoonoses such as anthrax, tuberculosis, brucellosis, salmonellosis, campylobacteriosis, and leptospirosis are widely distributed [4]. These infections are exacerbated by the socio-economic realities of rural areas. Close interactions between livestock and humans, including chickens, are a common scenario in daily rural life. Particularly, farmers, butchers, and live animal market workers are at risk due to frequent, unprotected contact with infected animals [5]. Table 1 displays the host, distribution, transmission, and case fatality rates of common transboundary and zoonotic diseases.

**Table 1.** Epidemiology of the most common transboundary and zoonotic diseases.

Disease	Type	Affected species	Distribution	Transmission	Case fatality	Annual Case	Ref.
Foot-and-Mouth Disease (FMD)	Transboundary	Cloven-hoofed animals	Global (esp. Africa, Asia, South America)	Direct contact, aerosols, fomites	High in outbreaks	N/A (Animal-specific)	[6]
Rift Valley Fever (RVF)	Zoonotic	Ruminants, humans	Africa, Middle East	Mosquitoes, contact with infected animals	1–10% (humans)	Variable; outbreaks of 10,000+ human cases	[7]
Avian Influenza (H5N1, H7N9)	Zoonotic/Transboundary	Poultry, wild birds, mammals	Global (notably Asia, Europe, Africa)	Direct contact with birds, contaminated environments	30–60% (H5N1)	Hundreds annually (humans)	[8]
Zika Virus	Zoonotic	Human, non-human primates, domestic animals, other animals	Americas, Southeast Asia, Africa	Mosquitoes (Aedes species), sexual transmission	Low (<1%)	500,000+ (2015–2016 peak)	[9]
Anthrax	Zoonotic	Human, domestic animals	Global (esp. Africa, Asia)	Contact with infected animals or spores	10–20% (cutaneous, untreated)	2,000+ human cases globally	[10]
Ebola Virus Disease	Zoonotic	Humans, wildlife	Sub-Saharan Africa	Contact with infected body fluids, bushmeat	25–90% (depending on outbreak)	1,000-30,000 (outbreaks)	[11]
African Swine Fever (ASF)	Transboundary	Pigs, wild suids	Africa, Asia, Europe	Direct contact, fomites, ticks	N/A	Millions of pigs annually (no human cases)	[12]
Brucellosis	Zoonotic	Human, domestic animals	Global (esp. Mediterranean, Asia, Africa)	Contact with infected animals, consumption of unpasteurized dairy	Low (<2%)	>500,000 human cases annually	[13]
Rabies	Zoonotic	Mammals, humans	Global (esp. Asia, Africa)	Bite of infected animals (e.g., dogs, bats)	~100% (if untreated)	59,000 human deaths annually	[14]
Bovine Tuberculosis	Zoonotic	Human, domestic animals	Global (esp. developing countries)	Aerosols, contact with infected cattle	8.1% approximately	140,000 cases worldwide in 2019	[15]

N/A-Not applicable; Ref. – references.

Accurate disease diagnosis is a key concern in medicine for effective treatment and the control of infectious disease spread [16]. Rapid diagnosis and intervention are vital to alleviate the detrimental effects of infectious diseases on animals, livelihoods, and national economies. Initial disease diagnosis involves clinical examination and medical history. Clinical assessments and medical history provide valuable insights, but many infectious diseases exhibit overlapping symptoms; therefore, laboratory testing is necessary for definitive diagnosis [17, 18]. Conventional diagnostic techniques, including staining, culture, and phenotypic characterization, such as biochemical tests, antibiotic susceptibility assays, and cytopathic evaluation of viruses in tissue cultures, remain widely used. Parasites are detected by microscopy or by immunoassays for antigens or antibodies. These old methods are reliable but time-consuming, not always sensitive, and require skilled workers to carry them out [19]. Insufficient veterinary services, substandard laboratory equipment, and logistical constraints pose significant challenges for low- and middle-income countries (LMICs) in animal health monitoring and diagnosis. These constraints frequently lead to delayed diagnosis, obstructing prompt action and disease management initiatives [20, 21].

In recent years, significant changes in diagnostic processes have enabled the rapid and accurate identification of pathogenic bacteria. Nowadays, molecular approaches are increasingly important for clinical diagnosis, therapeutic monitoring, and epidemiological studies. These new methods are slowly replacing older methods of diagnosis [22]. Advanced molecular techniques, like polymerase chain reaction (PCR) and its variants, enzyme-linked immunosorbent assay (ELISA), next-generation sequencing (NGS), and matrix-assisted laser desorption/ionization time-of-flight mass spectrometry (MALDI-TOF MS), are considered standard worldwide for their accuracy in diagnosing animal diseases [23]. These molecular and serological methods are especially significant for notifiable diseases, where specialized labs use tests recommended by the World Organization for Animal Health (WOAH) to confirm the diagnosis [24].

Molecular and serological procedures performed in a lab are known to be quite accurate. However, there are difficulties in use, as they require specialized facilities, skilled technicians, and robust logistical support, which can lower specimen quality and delay the availability of findings [25]. To overcome these problems, new diagnostic procedures have been developed that improve sensitivity, speed up results, and provide point-of-care (POC) capabilities. Lateral flow assay (LFA), loop-mediated isothermal amplification (LAMP), biosensors, and recombinase polymerase amplification (RPA) are some of the new technologies that can be used in the field to diagnose animal infections [23].

LFAs are rapid, simple, and portable ways of identifying diseases. They can detect DNA, RNA, proteins, biological agents, and chemical substances. Recently, LFAs have been employed in veterinary and biomedical settings for health monitoring, detecting infectious diseases, and assessing feed contamination [26]. In places with limited resources, such as least developed countries, where cold chain maintenance is sometimes impossible, they are invaluable. LFAs enable on-site diagnostics without a lab requirement. The application of LFAs reduces the need for sample movement and shortens the time to control diseases. LFAs help farmers act quickly by providing rapid, accurate findings. This stops the spread of disease and ensures that sick animals get the medication they need on time. These traits make LFAs a game-changing tool for animal disease diagnosis, especially in areas with limited veterinary support or restricted infrastructure. This review paper demonstrates the importance of LFAs for rapid testing and their potential to improve disease control and protect public health.

## 2. History of the lateral flow assay

LFAs are often used to detect specific analytes in complex mixtures [27]. Since their emergence in the 1980s, LFAs have undergone significant changes driven by advances in immunology, materials science, and point-of-care diagnostic technologies. In the 1960s and 1970s, the development of immunoassays, particularly the enzyme-linked immunosorbent assay (ELISA), was the root of LFA’s invention. Though ELISA has high sensitivity and specificity, it requires a designated laboratory and time to process, making it difficult to use without a centralized location [28]. This led scientists to make immunoassays easier to use so that more people might use them, especially in field and point-of-care settings.

The first LFAs emerged in the 1980s, in which liquid samples were transferred through a porous membrane by capillary action. The assay design was inspired by earlier strip methods, such as dipstick assays for measuring pH and glucose in urine [26]. The application of antibodies or antigens to the strip was a game-changer for lateral flow technology. Detection of specific target analytes became possible due to this integration. The first commercial LFA was developed using a specific antibody to detect human chorionic gonadotropin (hCG) in urine during pregnancy. This early test demonstrates the feasibility of quick, one-step diagnostics without specialized tools [29].

During the 1990s, significant improvements in LFAs were driven by the development of effective antibodies, improved membrane materials, and variations in labeling [30]. The addition of monoclonal antibodies made LFAs more specific and sensitive. Nitrocellulose membranes were found to be the best material due to their excellent binding and capillary flow properties [31]. A number of LFAs have been developed for infectious disease detection, such as malaria, human immunodeficiency virus (HIV), and the flu, as well as for testing drugs and monitoring the environment [32]. The addition of different labels, such as colloidal gold, latex beads, and fluorescent dyes, improved the precision and accuracy of these tests [33].

In the 2010s, a lot of research was conducted to improve LFA’s performance and expand its capabilities. Nanotechnology offers opportunities to use new nanomaterials, such as gold nanoparticles, quantum dots, and carbon nanostructures, which greatly enhance signal amplification and sensitivity [34]. The application of smartphone-based readers and connected microfluidic devices has also changed LFAs into semi-quantitative or quantitative diagnostic tools. The gap between traditional quick tests and laboratory-based procedures was filled [35].

**Figure 1.**
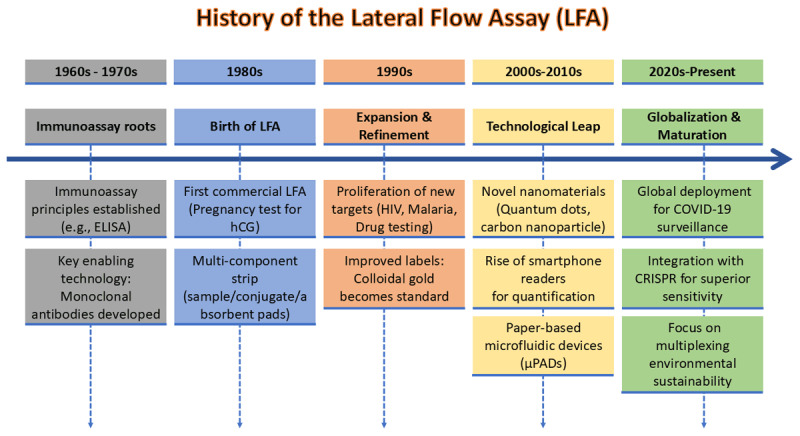
Illustration of the timeline of the lateral flow immunoassay development.

The COVID-19 pandemic (2020–2022) demonstrated the importance of LFAs worldwide as essential tools for mass testing and surveillance. Rapid antigen testing (a form of LFA) has become a key part of pandemic response plans worldwide, as it is simple to use, inexpensive, and can provide results in minutes [36]. This time also led to new ideas in multiplexing, enabling the simultaneous detection of multiple analytes on a single strip. Figure 1 represents the key milestones in the development timeline of the lateral flow immunoassay.

LFA has advanced from simple multi-component structures to single paper-based microflow analysis devices (µPADs), in which hydrophobic channels are patterned on a single sheet to guide fluid flow via capillary action. Sample processing, reagent storage, and detection are integrated into a compact, instrument-free platform, reducing material costs and improving portability. These trends are driven by the demand for higher sensitivity, multiplexing capabilities, and integration across various applications, particularly in point-of-care diagnostics. [26, 30, 37, 38, 39, 40]. The analytical sensitivity and selectivity of the LFAs also improved significantly with the incorporation of new nanomaterials as signal transducers or receptor immobilization supports [27]. Approaches to improve the assay performance also involve improvement in amplification systems, recognition entities, and design formats [19]. Typical LFAs were generally restricted to single-analyte detection. Nevertheless, multiplex lateral flow assays enable the detection of multiple targets in a single sample, thereby improving diagnostic precision and efficiency. In contrast, it reduces the required reagent volume and sample volume. LFAs remain at the core of PoC diagnostics due to their simplicity, quick turnaround time, user-friendliness, cost-effectiveness, and disposability [19, 26, 30].

Recent advancements in LFAs aim to enhance sensitivity to competing molecular diagnostics such as PCR [41]. Researchers are actively exploring techniques, including signal amplification, machine learning-based analysis, and the use of CRISPR-based technologies [26, 42, 43]. Also, LFAs are being modified to support new applications, such as identifying cancer biomarkers, ensuring food safety, and tailoring treatment.

## 3. Overview of LFA

### 3.1. Principles of LFA

**Figure 2.**
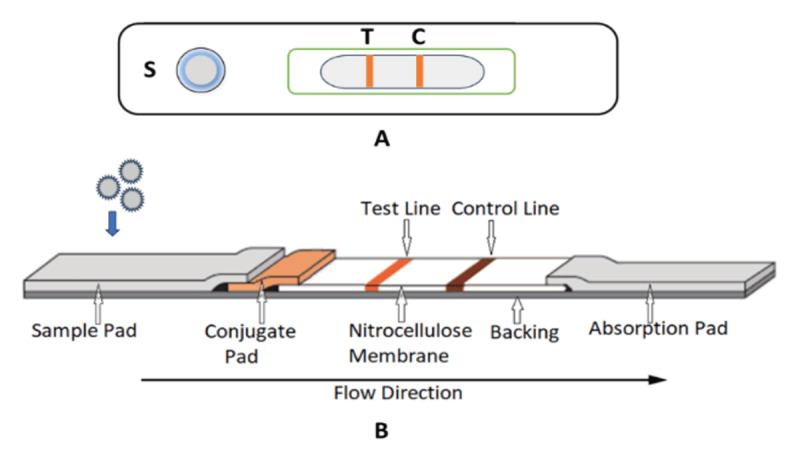
(A) Front view of a lateral flow immunoassay strip. (B) Schematic representation of the lateral flow immunoassay mechanism.

Lateral flow assays are self-operating, capillary-driven diagnostic devices designed for rapid and point-of-care use. Based on the principle of immunochromatography, the assays comprise prefabricated strips embedded with dry reagents, which are activated upon the addition of a fluid sample. The fundamental principle parallels that of enzyme-linked immunosorbent assay (ELISA), as both methods rely on specific molecular recognition between a target analyte and its complementary biorecognition element, such as an antibody, aptamer, or nucleic acid probe. LFAs exhibit high affinity and specificity of molecular recognition, such as antigen-antibody, aptamer-protein, or probe-nucleic acid interactions, for detecting trace levels of analytes, including proteins, peptides, hormones, and antibodies, in complex biological fluids. Signal generation for detection is achieved through detection labels, most commonly colored nanoparticles such as gold, latex, or carbon in colorimetric LFAs and fluorescent, surface-enhanced Raman scattering (SERS), or electrochemical labels, which are increasingly used in quantitative applications. The labels are pre-bound to primary bio-recognition molecules and form complexes such as antibody-nanoparticle or aptamer-nanoparticle complexes, also known as conjugates. Upon rehydration, these conjugates bind selectively to the target analyte, forming a label-analyte complex as the sample migrates by capillary action. The complex binds to a secondary immobilized bio-recognition element at the test line, producing a signal that can be visualized or quantitatively analyzed with appropriate readers [41, 42, 43, 44, 45, 46, 47, 48]. Figure 2 illustrates the lateral flow immunoassay strip and its various components.

During the test procedure, a liquid sample is placed on the test equipment’s sample application pad, which acts as a prefilter, removing large debris and regulating sample pH and flow rate. The sample migrates laterally across a porous sample pad to the conjugate pad by capillary action. The conjugate pad contains dried detection conjugates that bind selectively to target analytes, forming a label-analyte complex. The complex moves forward via capillary action and is captured at the test line on the nitrocellulose membrane by a secondary immobilized biorecognition element, thereby generating a signal. The presence or absence of a signal determines test results. Additionally, the appearance of a separate control line confirms fluid migration, reagent functionality, and test validity for both positive and negative results. Finally, the absorbent pad maintains unidirectional flow by wicking away surplus fluids. The combination of precise biorecognition and sensitive labelling technology enables LFAs to provide reliable, instrument-free, and rapid diagnostics suitable for clinical, environmental, and field-based testing [49, 50].

### 3.2. Component of LFA

The main components of LFA are the conjugate pad, sample pad, biorecognition elements, labels, membrane, and absorbent pad. The pads and membrane are assembled onto a backing card to create the test strip. The strip is housed on a cassette [32, 51].

### 3.3. Sample pad

The sample pad serves as the application pad for samples. The pad is composed of cellulose or glass fiber. The selection of material affects the pad’s compatibility with the testing objectives and sample properties. The pad is incorporated with various substances, including proteins, detergents, viscosity enhancers, and buffer salts for pretreatment of the sample prior to further processing along the LFA strip [52]. Additives can segregate components, remove interferences, and alter the ionic conditions, viscosity, and chemical composition of the sample. The viscosity of the sample can be increased to extend reaction times, or target compounds can be chemically modified to enhance binding at the test line. A symmetrical or asymmetrical arrangement of the pad’s pores acts as an initial filter. Coarse materials, such as whole cells, can be trapped within the pad depending on the distribution of pore sizes. The sample pad makes it easier to evenly spread the sample solution onto the conjugate pad, ensuring a consistent application [32, 51, 53].

### 3.4. Conjugate pad

The conjugate pad is typically made from polyesters, glass fibers, or cellulose. It contains a dried, immobilized primary biorecognition element, such as a labeled antibody complex, which is crucial for detecting the target analyte. Upon contact with the sample diluted in buffer, these labeled molecules dissolve into the fluid. The formation of the label-analyte complex through interactions between the labeled antibodies and the target analyte continues throughout the chromatographic process. An ideal conjugate pad minimizes nonspecific binding and ensures consistent flow characteristics for smooth and uniform movement. It requires a minimal volume of labeled conjugate, is free from loose particles that could block the membrane capillaries, and facilitates efficient detection [53, 54].

### 3.5. Labels

The labels are essential for improving sensitivity and detection in the analytical processes of lateral flow assays, as they aid in signal detection. Labels are typically composed of colored nanoparticles or enzymes. Common types of nanoparticles are colored latex beads, colloidal gold, quantum dots, and magnetic nanoparticles, as well as more recent developments in carbon, silica, and europium nanoparticles. Advanced lateral flow assay formats may also employ fluorescently labeled liposomes, Raman-active tags, or fluorophores. The choice of label depends on assay requirements, including colloidal stability, binding characteristics, detection limits, multiplexing capabilities, availability, and cost. In device reader systems, different types of fluorescent assays are used. In contrast, visual assays commonly employ gold, colored latex, and carbon labels. Among these, gold nanoparticles are the most widely used due to their strong affinity for antibody binding, vibrant color, and high stability in optimized assays [19, 55, 56, 57, 58].

### 3.6. Membrane

The membrane is the most critical element in LFA, used as a strip material and made of porous materials such as nitrocellulose, cellulose acetate, polyether sulfone, or nylon. Due to its low cost, excellent protein adsorption, and tunable wicking properties, nitrocellulose is a widely used LFA membrane [53]. The membrane is a suction over water, and the porous sponge structure has a pore size range of 0.05–12 μm [59]. Its high capacity to bind and immobilize proteins and exhibit capillary forces is advantageous for assay performance. Nitrocellulose membrane facilitates the binding of target molecules and allows interaction between the sample solution and immobilized reagents, e.g., antibodies or aptamers. It contains immobilized antibodies, proteins, or antigens in lines or spots as test and control indicators for detecting the target analyte and ensuring assay validity [27, 60].

### 3.7. Absorbent pad

The absorbent pad is the last portion of the strip, acting as a sink to wick excess liquid from the membrane and prevent sample backflow. The absorbent pad markedly decreased nonspecific binding, thereby enhancing the assay’s overall sensitivity. Most often, cellulose filters are used as pad material [52].

### 3.8. Mechanism of nitrocellulose membrane/other cellulose-based paper materials

#### 3.8.1. Capillary action

A nitrocellulose membrane enables liquid flow via capillary forces, which is essential for reagent transfer. The contact time, the sensitivity, and the specificity of the assay depend on pore size [61].

#### 3.8.2. Protein adsorption and immobilization

Nitrocellulose can absorb protein with ionic strength dependent on electrostatic and hydrophobic interaction forces [53]. This immobilizes reagents, e.g., antibodies, on test and control lines.

#### 3.8.3. Pore size filtration

Pore architecture regulates the passage of larger particles, thereby minimizing nonspecific binding of substances other than the target analyte [59]. Furthermore, specialized membranes can separate plasma from blood cells.

#### 3.8.4. Selective binding by immune reaction

Selective detection is achieved by specific binding of immobilized capture reagents (e.g., antibodies) to the target analytes. Non-analyte material passes through to the membrane and is taken up by the wicking pad.

#### 3.8.5. Reduction of nonspecific interactions

Blocking reagents are used to reduce the formation of false positives by binding to non-target sites on the membrane, thereby preventing interactions that are not true positives [61].

#### 3.8.6. Other cellulose-based paper materials

Although nitrocellulose is the most commonly used paper substrate in LFAs, other cellulosic paper-based materials are being studied owing to their cost-effectiveness, recyclability, and ability to keep bioreceptors dry [62]. These materials have approximately comparable filtration and flow properties: capillary action for fluid transport and filtration on the basis of their fibrous network structure as well as pore size [63].

Scientists are trying to tweak cellulose paper so it does more. For example, antibodies and cellulose papers can be modified by the presence of carbohydrate-binding module (CBM)- fused antibodies to increase protein-binding capacity and LFA sensitivity. Surface functionalization can also reduce nonspecific adsorption, thereby enhancing assay specificity [64].

### 3.9. Formats of LFA

**Figure 3.**
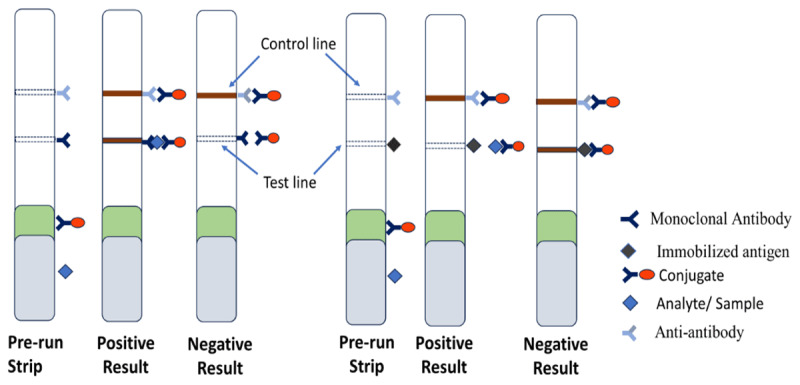
Analyte detection of sandwich and competitive assay formats. The appearance of the Test line indicates a positive result in the sandwich assay and a negative result in the competitive assay.

There are different LFA formats based on the type of target analyte. The most frequently used formats are sandwich assays and competitive assays [65]. Figure 3 shows the different LFA formats based on the type of target analyte.

#### 3.9.1. Sandwich assay

The sandwich assay is a non-competitive, or direct, assay format for high-molecular-weight analytes with multiple antigenic determinants, such as viral proteins. This assay employs two different antibodies: a capture antibody and a detector antibody. The antibodies bound to certain parts of the analytes. A tagged antibody on a conjugate pad served as the detection reagent, while a monoclonal antibody on the test line of the nitrocellulose membrane served as the capture reagent. A colored line at the test location shows a positive result because the analyte is trapped between the detection and capture antibodies, making a sandwich. The reaction in the capture zone is directly related to how much of the analyte is in the sample [66, 67, 68].

#### 3.9.2. Competitive assay

The competitive assay in inhibitory form is designed for analytes characterized by low molecular weight and a singular antigenic site, encompassing small organic molecule pharmaceuticals, steroids, and various other substances. The assay can be qualitative, semi-quantitative, or quantitative. In this format, the conjugate pad contains a prefixed-labeled antibody or aptamer, and the test line on the nitrocellulose membrane contains a pre-immobilized antigen, usually a protein-analyte complex that binds specifically to the labeled conjugate. If the target analyte is in the sample, it sticks to the labeled conjugate and prevents it from sticking to the analyte in the test line. When the target analyte is not present or is present in low amounts, the pre-immobilized antigen will connect with the labeled conjugate at the test line, making a signal. The amount of analyte in the sample is inversely related to the signal strength [4].

#### 3.9.3. Multiplex assay

**Figure 4.**
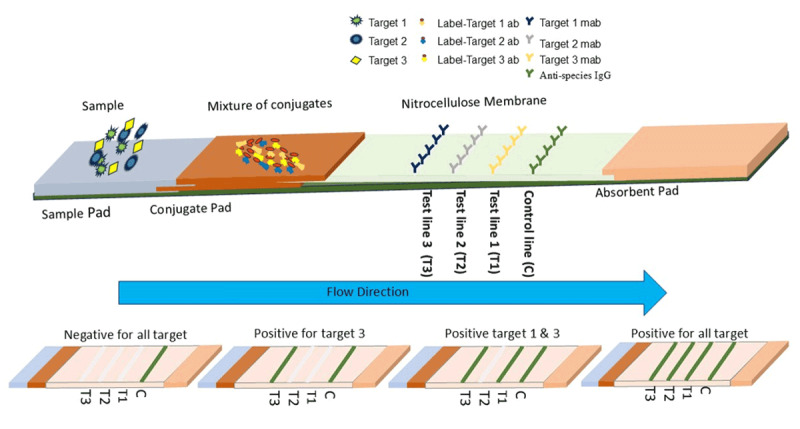
Schematic diagram of a multiplex lateral flow immunoassay (LFIA) to simultaneously analyses multiple targets in line formats.

**Figure 5.**
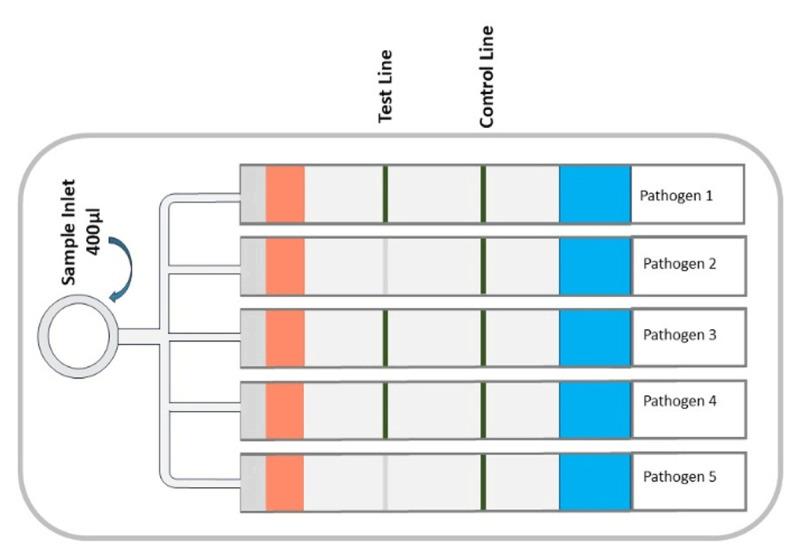
Schematic diagram of integrating multiple individual test strips into a single device cassette for the simultaneous processing of samples.

Detection of multiple biomarkers at once, differentially diagnosing diseases, or identifying specific agents from several suspected agents by running a single assay can avoid the result variations from individual run assays. The concept is used in multiplex lateral flow assays, where the whole process is simplified by reducing sample volume, time, and cost. Multiplex LFA uses advanced technology to find more than one analyte in a single sample at the same time with just one strip. Popular strategies for multiplexing lateral flow immunoassays are (a) integrating several analyte test lines or dots (Figure 4) [69], (b) Conjugating various colors or multi-fluorescence signals, such as dyes, quantum dots, and upconverting phosphors, with a recognition element and (c) integrating multiple individual test strips into a single device cassette for the simultaneous processing of samples (Figure 5) [69, 70, 71]. A successful example of this type of multiplex lateral flow disk that utilizes up-converting phosphor technology and features 10 detection channels can identify 10 different foodborne bacteria [72].

#### 3.9.4. Single paper-based µPADs

The evolution of multicomponent LFAs toward single-paper µPADs is conceptually and functionally a big leap [38, 39]. In µPADs, the flow of fluids by capillarity is conducted through hydrophilic zones and hydrophobic barriers on paper, which is printed with micro-channels. This permits advanced multidirectional microfluidic elements and more sophisticated analytical operations [40]. µPADs enable complex fluid handling procedures such as transport, sorting, mixing, and splitting all in one assay. This increased level of control supports more complex bioanalytical assays, resulting in multiplexing dozens of tests into a single device. Several methods, such as photolithography, wax printing, inkjet printing, micro-plotting, screen printing, and laser engraving, are used for the preparation of µPADs [40]. The use of nanomaterials in µPADs has significantly improved signal response, previously limited by sensitivity and specificity for paper-based analytical devices [38].

### 3.10. Advantages and disadvantages of LFAs

LFAs are widely utilized diagnostic tools in medical, environmental, and food testing. These assays are designed to detect specific substances, such as pathogens, biomarkers, or contaminants, in a sample, typically employing a simple strip-based format [73, 74].

#### 3.10.1. Advantages

##### 3.10.1.1. Speed and efficiency

Simple test procedure with less analysis time. Sensitive to proteins, haptens, and nucleic acid amplicons. It produces visual and easily interpreted results with moderate to high sensitivity and specificity. Detection limits are between 0.1 and 10 ng/ml with a low constant of variance (CV) in both competitive and noncompetitive formats. Demonstrate qualitative (present/absent) or semiquantitative results.

##### 3.10.1.2. Cost-effectiveness

Easy to use, one-step rapid test, inexpensive, lightweight, portable, and low-energy-consumption. Individual tests or, at times, array formats for batch-wise mid-throughput screening. The potential of multiplexing.

##### 3.10.1.3. Easy sample preparation

Pretreatment of fluid samples is often not necessary for the application; there is no need for washing stages. Required a small volume of sample, appropriately applied to the assay without contamination.

##### 3.10.1.4. Convenience/decentralized POC diagnostics

The process is accessible and does not require specialized qualifications. Applicable for use by general practitioners or patients in a home setting. Suitable to use at the point of care/need, whether in human health, veterinary diagnostics, the food industry, or disease surveillance. Single-use and disposable properties eliminate contamination possibilities from previously tested samples.

##### 3.10.1.5. Minimal barriers to market

Relatively easy manufacturing procedures and reduced development time bring applications faster to the market and have high commercialization potential. Long shelf life/environmental stability (1–2 years) at room temperature; larger batches can be produced to reduce variation between batches.

#### 3.10.2. Disadvantages

##### 3.10.2.1. Result interpretation

Subjective result interpretation. Mainly qualitative or semi-quantitative. Positive results may require confirmatory analysis. Reproducibility may vary from batch to batch. Under a competitive structure, signal intensity is inversely proportional to analyte concentration.

##### 3.10.2.2. Sample handling

Solid samples require pretreatment. Sample properties (viscosity and surface tension) affect analysis time. Inaccurate sample volume causes decreased precision.

##### 3.10.2.3. Preparation challenges

A high-quality antibody preparation or hybridizing nucleic acid sequence is essential. Poor biomolecular affinity for analytes and a tendency for cross-reactivity. It is not possible to enhance responsiveness through an enzyme reaction. Matrix components facilitate the closure of membrane gaps.

## 4. Role of rapid test kits in livestock disease diagnosis

Veterinary medicine focuses on preventing, controlling, diagnosing, and treating diseases in both domestic and wild animals, while also working to prevent the spread of diseases to humans [75, 76]. It adequately addresses the diversity of animal species. Veterinarians provide health care for a wide range of animals, including pets like dogs and cats, farm animals like cows, lambs, chickens, and pigs, as well as wild animals, zoo animals, and aquatic species [77, 78].

The World Organization of Animal Health (WOAH) and the Food and Agriculture Organization (FAO) had prioritized animal health, food safety, and veterinary public health, as animals play a vital role in food security, sustainable agriculture, and disease prevention [79]. Animal diseases, especially transboundary animal diseases (TADs), spread quickly among livestock, making it challenging to trade animal products around the world. They stop live animals and animal products from moving. This situation poses serious socio-economic threats with severe consequences [80].

LFA rapid tests were routinely employed to detect viral antigens and antibodies (IgG and IgM) in patients during outbreaks of severe acute respiratory syndrome (SARS), Middle East respiratory syndrome (MERS), and SARS-CoV-2 (COVID-19). These tests were crucial for identifying infected individuals, asymptomatic cases, and individuals with varying degrees of immunity [81]. A simple, disposable, rapid antigen-detection test was invaluable in eliminating the last remnants of infection during the rinderpest eradication efforts in Pakistan and Somalia [82].

Due to their widespread distribution and high mortality rates, avian influenza viruses (AIVs) pose global challenges. In the past 20 years, highly pathogenic avian influenza (HPAI) has caused many avian outbreaks. From the year 2003 to 2023, the WHO recorded 878 cases of HPAI H5N1 infection in humans in 23 countries with a 52.16% death rate [83]. Rapid antigen testing for HPAI was an effective method for early identification and containment in this period [84].

Efficient monitoring, prompt identification, transparency, and swift detection are essential elements for the prevention and management of animal disease outbreaks. Immediate disease diagnosis facilitates better management and intervention strategies [85]. Studies indicate that veterinarians use rapid tests to assess various medical issues in both agricultural animals and domestic pets. The identification of infectious diseases accounts for 93% of all lateral flow immunochromatographic assay (LFIA) applications, of which 51% are viral, 27% bacterial, and 22% other pathogenic infections. Drug detection is the second most prevalent use. In instances where culture is challenging and serology may be complicated by prior vaccinations, rapid tests are often the best choice [86].

LFIAs for veterinary use must be able to work with a wide range of animal species and their unique traits. They must also be able to work with different types of samples, such as serum, urine, mammary secretions, milk, buccal and nasal secretions, feces, and respiratory exhalations [86]. The increasing incidence of transboundary and zoonotic diseases in livestock and companion animals, along with the growing need for rapid disease diagnosis, is propelling the veterinary sector to advance these assays. The expanding number of companion animals and the rising demand for animal-derived food items are key drivers of market expansion [87].

In 2023, the global LFA market was worth $10.8 billion. By 2029, it is expected to be worth $18.8 billion, which means it will increase at a rate of 7.6% per year. The worldwide veterinary quick tests market is projected to expand from $942.3 million in 2024 to $2.87 billion by 2034 [88]. Prominent entities in the veterinary rapid tests sector comprise IDEXX Laboratories, Inc. (US), Heska Corporation (US), Fassisi GmbH (Germany), BioNote, Inc. (South Korea), MEGACOR Diagnostik GmbH (Austria), Zoetis, Inc. (US), Biopanda Reagents Ltd. (UK), Virbac (France), SWISSAVANS AG (Switzerland), and Woodley Equipment Company (UK) [89].

## 5. LFA in aquaculture

LFA has been increasingly used to detect various fish and shrimp pathogens in aquaculture farms due to its minimal device size, ease of use, and simple readout mechanisms. Zheng et al. [90] developed and tested an LFIA for the on-site detection of Cyprinid herpesvirus 3 (CyHV-3), which is the virus that causes koi herpesvirus disease (KHVD) and has had a significant impact on the ornamental and food-producing carp industries. They use colloidal gold to connect to the CyHV-3 antigen, and the lowest limit of detection was 1.5 × 10^4^ copies/μl with 100% specificity. The Commercial WSSV RP Rapid Test Kit (WSSV RP Rapid Test Kit, Innocreate Bioscience Co., Ltd., Taiwan) was developed to find White Spot Syndrome Virus (WSSV), a very contagious virus that poses a serious threat to shrimp farming around the world. The test kit has a sensitivity of 99.21% and a specificity of 100% when compared to the TaqMan real-time PCR method [91]. WOAH has approved the kit for quick WSSV detection. A different immunochromatographic test strip was made to find the fish pathogen *Edwardsiella tarda* quickly. It used a monoclonal antibody conjugated with colloidal gold as a detector. The limit of detection (LOD) was 1 × 10^5^ colony-forming units (CFU/ml) [92], which was quite accurate and didn’t cross-react. The LFIA developed by Wangman et al. [93] can detect approximately 40 ng/ml of PirB toxin from Vibrio species responsible for acute hepatopancreatic necrosis disease (AHPND) within 15 min of applying either bacterial colonies or pre-enrichment samples.

## 6. LFA in livestock and poultry

### 6.1. LFA in livestock

FMD is a highly contagious viral disease that affects domestic animals. Effective control of this disease requires quick, sensitive, and specific diagnostic tools at every level of the control strategy. Several rapid, easy-to-perform tests, including on-site LFAs, have been developed to detect suspected FMD outbreaks and serotype [94]. One such lateral flow device (LFD), created using the monoclonal antibody Mab 1F10, can detect FMDV across all seven serotypes. The diagnostic sensitivity of this LFD is 84%, compared to 85% for the reference antigen ELISA, and the diagnostic specificity is around 99%, compared to 99.9% for the reference method [95]. Another gold immunochromatographic strip test kit can detect FMDV types O, A, and Asia I, achieving sensitivity rates of 86.9%, 83.8%, and 92.01%, respectively, with a specificity of 100%. The overall accuracy for detecting FMDV serotypes with this kit is 94.58% [96].

In non-laboratory environments, an LFA using the monoclonal antibody MAb 70–17 may also detect four FMDV serotypes (O, C, A, and Asia 1). This assay has a diagnostic sensitivity of approximately 87.3% and a specificity of 98.8%, compared to 87.7% and 100% achieved with the antigen-ELISA [97]. The lateral flow strip utilizing monoclonal antibodies (MAbs) for FMDV serotyping can detect all seven serotypes and differentiate between serotypes A, O, C, and Asia 1, with sensitivities ranging from 10^3^ to 10^4^ TCID_50_ doses of each FMDV strain [98]. The VDRG^®^ FMDV 3Diff/PAN Ag Rapid Kit, manufactured by MEDIAN Diagnostics Inc., has been shown to function as a lateral flow test or pen-side test for detecting FMDV serotypes A, O, and Asia-1.

PPR is a highly infectious viral disease that impacts tiny ruminants and wild artiodactyls. It is a notifiable disease recognized by the WOAH and is considered transboundary. The FAO and WOAH have prioritized PPR for global eradication, aiming to achieve this goal by 2030 [99]. Using superficial swab samples (ocular or nasal), an immunochromatographic test can identify all four known PPR genetic lineages, even in cases with only minor clinical symptoms, starting 4 days post-infection. When tested against PCR, this test has a sensitivity of 84% and a specificity of 95% with a detection limit of 10^3^ TCID_50_ (50% tissue culture infectious doses). This diagnostic instrument might greatly help present initiatives to stop the PPR spread [100, 101].

Lumpy skin disease is a vector-borne disease that affects Asian water buffalo and cattle. In 2019, it spread in China, India, and Bangladesh and, subsequently, in Nepal and Bhutan [102]. The rapid spread of this disease necessitates the development of effective control strategies, which rely on rapid diagnostic tools to prevent outbreaks. A highly sensitive LFA was developed using monoclonal antibodies (MAbs) targeting LSDV structural protein p32 and gold nanoparticles (AuNPs). The kit demonstrated a lower detection limit of 103.4 TCID_50_/ml, along with satisfactory inter- and intra-assay repeatability (less than 5.3% coefficient of variation [CV%]) and confirmed specificity [103]. A second gold nanoparticle lateral flow test (GNP-LFT) was also made using polyclonal antibodies against LSDV, demonstrating it is as sensitive and specific as ELISA in finding samples from infected animals [104]. These improvements show that LFA is a valuable technique for diagnosing LSD at the time of need.

Rift Valley Fever (RVF) is a zoonotic disease caused by the *Phlebovirus* and spread by mosquitoes. The disease is common in Africa, the Arabian Peninsula, and some islands in the Indian Ocean. RVF is associated with elevated abortion rates, neonatal mortality, fetal abnormalities, abrupt fatalities in ruminants, and varied severities of sickness in humans.

Strict biosafety and biocontainment measures are required for the diagnosis of RVF in traditional serological and molecular techniques. [105]. In resource-constrained situations, there is an urgent need for fast and precise assays for on-site identification of the RVF virus and bolstering effective mitigation strategies. Cetre-Sossah et al. [106] developed a lateral flow strip test targeting the rNp protein of RVFV. In validation, the assay demonstrated 100% diagnostic sensitivity and 98.81% specificity against isolates from different hosts and geographic regions. The WOAH recommends this kit as a first-line diagnostic tool for RVFV antigen detection, provided that it is used with appropriate personal protective equipment. Domfe et al. [107] designed an antibody detection LFA capable of detecting a minimum concentration of 0.125 mg/ml antibody against RVFV. Another LFA was able to identify 95% of 1:128 serum dilutions from vaccinated sheep, underscoring the importance of LFA for detection and sero-monitoring [108].

All mammals, including humans, are susceptible to rabies, the oldest known deadly zoonotic disease that is brought on by lyssaviruses, which are primarily transmitted through bites from infected animals, which introduce infectious saliva [109]. Approximately 59,000 people die from rabies each year, averaging one death every nine min. Around 40% of the rabies victims are children, and 95% of cases occur in developing countries of Asia and Africa. A worldwide project seeks to eradicate human fatalities resulting from dog-mediated rabies by 2030 [110]. The direct fluorescent antibody (DFA) test, direct rapid immunohistochemistry test (dRIT), and reverse-transcription polymerase chain reaction (RT-PCR) assay enable a reliable diagnosis of rabies. Accuracy ratings on these tests range from 98% to 100%. Furthermore, positive results from LFA should strongly indicate the need for post-exposure prophylaxis for those exposed [111]. Evaluations of commercial rapid immunochromatographic tests for rabies diagnosis have shown sensitivity above 94% and 100% specificity when compared to DFA and dRIT tests using brain samples and nuchal skin biopsies [112, 113, 114]. These findings suggest that LFA is a practical tool for improving reporting and enhancing surveillance in resource-limited settings.

### 6.2. LFA in poultry

Newcastle disease virus, avian influenza virus, infectious bursal disease virus, and infectious bronchitis virus are all major viral infections that cost the chicken business a lot of money. Meseko et al. [115] found that the relative diagnostic sensitivity was 84.3% (95% confidence interval [CI], 78.1–88.9%), and the relative diagnostic specificity was 97.7% (95% CI, 94.2–99.1%) when they tested a commercially available antigen fast AIV Ag test kit in Nigeria to see if it could identify HPAI. Fast immunochromatographic assays for detecting the avian influenza A (H7N9) virus use anti-hemagglutinin monoclonal antibodies conjugated with 0.01% colloidal gold and show no cross-reactivity to other avian influenza A subtypes.

These assays demonstrated a detection limit of 10^3.5^ pfu/ml, or 10^3^ TCID_50_, reported by Kang et al. [116]. Li et al. [117] assessed an immunochromatographic strip designed for detecting NDV. The strip utilized anti-hemagglutinin-neuraminidase monoclonal antibodies labeled with colloidal gold. Polyclonal antibodies from chickens that fight NDV and staphylococcal protein A were used as test and control lines on the nitrocellulose membrane. RT-PCR confirmed that this strip has a diagnostic sensitivity of 83.3% and a specificity of 100%. It also had a detection limit of 10^4.9^ EID_50_ viruses per 0.1 ml in NDV-infected samples, and it didn’t react with other viruses.

### 6.3. LFA in companion animals

Companion animals, such as pets, play a vital role in human social life by significantly contributing to both physical and mental health. They provide companionship, reduce stress, alleviate loneliness, and encourage physical activity through interaction and caretaking routines. Still, companion animals may also cause infectious diseases in people and food-producing animals [118]. Zoonotic diseases may be transmitted through bites, scratches, or direct contact with animal tissues or fluids; indirect contact with contaminated objects or surfaces; airborne droplets; or arthropod vectors. Important diseases related to companion animal health and zoonosis include brucellosis, influenza, mycobacteriosis, rabies, salmonellosis, trypanosomiasis, and leishmaniasis [119]. To facilitate early diagnosis and effective control measures, lateral flow kits have emerged as significant tools in companion animal health. LFAs are currently available for a range of conditions, including *Giardia, Ehrlichia, Cryptococcus*, Lyme disease, feline panleukopenia virus (FPV), feline immunodeficiency virus (FIV), and canine parvovirus (CPV) [120].

LFAs are commonly used in veterinary and aquatic medicine due to their rapid results, user-friendliness, and adaptability to field conditions. Table 2 below lists notable LFAs used to diagnose veterinary and aquatic diseases, organized by target species and disease type.

**Table 2.** A list of notable LFAs used in diagnosing veterinary and aquatic diseases, organized by target species, along with their sensitivity and specificity.

Sl.	Name of kit	Manufacturer	Description	Analyte	Host	Sample type	Sensitivity /LOD	Specificity	Ref.
1.	Active Anthrax Detect™ Plus Rapid Test	Inbios, USA	Anthrax	Ag	Animal	Tissue, rectal swab, nasal swab.	82%	96%	[121]
2.	AIV H5 Ag Test	Ringbio, Chaina	Avian influenza H5	Ag	Poultry	Oropharynx, spleen, kidney of chicken	96%	100%	[122]
3.	Bovine Brucella Antigen Rapid Test	Rapidlabs, UK	Bovine brucella antigens	Ag	Cattle	Whole blood/serum/plasma	95.45%	97.70%	[123]
4.	BVDV Ag Point-of-Care Test	IDEXX, USA	Bovine viral diarrhea virus (BVDV)	Ag	Cattle	Ear-notch tissue Whole blood/serum/plasma	96%	98.5%	[124]
5.	Fassisi BoDia	Fassisi GmbH, Germany	Rotavirus	Ag	Calves	Feces	96.15%,	98.48%	[125]
			Coronavirus				90.91%,	98.77%	
			*E. Coli* K99				90.00%,	98.78%	
			Cryptosporidia				97.92%,	97.73%	
6.	Fassisi Corona	Fassisi GmbH, Germany	Canine coronavirus Feline coronavirus	Ag	Cats and dogs	whole blood, plasma or serum of infected dogs and horses	99.99%	97.50%	[126]
7.	FASTest^®^ AIV Ag	MEGACOR Diagnostik, Austria	Avian Influenza Virus Type A antigen	Ag	Birds	swab samples from cloaca, trachea, kidney or feces	100%	100%	[127]
8.	FASTest^®^ ANAPLASMA	MEGACOR Diagnostik, Austria	Anaplasma phagocytophilum Anaplasma platys	Ab	Dog and horse	whole blood, plasma or serum	99.0%	96.4%	[128]
9.	FASTest^®^ CRYPTO	MEGACOR Diagnostik, Austria	*Cryptosporidiu*m spp.	Ag	Pocket pets, pets and farm animals	Feces	96.7 %	99.9 %	[129]
10.	FIPV Rapid Test Kit	Ringbio, Chaina	Feline Infectious peritonitis virus	Ag	Feline	Serum, Blood	100%	>99%	[130]
11.	ID Rapid^®^ PPR Antigen	Innovative Diagnostics, France	PPR Virus	Ag	Ovine, caprine	ocular swabs	100%	>99%	[131]
12.	ID Rapid^®^ Rift Valley Fever Antigen	Innovative Diagnostics, France	Rift Valley Fever Virus (all Strain)	Ag	Cattle, small ruminants, camelids	Whole blood, plasma or serum	3.5×10^3^ PFU	99.5 %	[132]
13.	Rapid Bovine TB Ab	Bionote, Korea	Mycobacterium bovis	Ab	Bovine	serum, plasma, whole blood	81.7%	91.4%	[133]
14.	Rapid CDV Ag	Molecular Diagnostic Services, Africa	Canine Distemper Virus	Ag	Canine	Plasma, serum, urine, salvia or conjuctiva swab	97%	96.5%	[134]
15.	Rapid *E. canis* Ab	Molecular Diagnostic Services, Africa	*Ehrlichia canis*	Ab	Canine	Whole blood, plasma or serum	98.80%	98%	[135]
16.	Rapid NDV Ag	Bionote, Korea	Newcastle Disease Virus (NDV)	Ag	Chicken	Oropharynx, spleen, kidney	94.7%	96.4%	[136]
17.	Rapid Rabies Ag	Bionote, Korea	detection of rabies virus antigen	Ag	Canine, bovine or raccoon dog	fresh brain tissue	96.9%	100%	[137]
18.	Speed Duo Diro/Leish K	Virbac, France	Leishmania infantum kinesins	Ab	Dog	whole blood, serum, plasma	98%	100%	[138]
			*Dirofilaria immitis*	Ag			95.2%	99%	
19.	Speed Trio FeLV/FIV/Corona	Virbac, France	Feline Leukemia Virus (FeLV)	Ag	Cat	whole blood, serum or plasma	94.70%	99.20%	[139]
			Feline immunodeficiency virus (FIV)	Ab			96.30%	98.90%	
			Feline corona virus (FCOV)	Ab			96.50%	100%	
20.	Swine Fever Rapid Test	Rapidlabs, UK	Classical swine fever virus (CSFV)	Ab	Pig	Serum/plasma	97.50%	96.67%	[140]
21.	VDRG^®^ ASFV Ag Rapid kit	Median diagnostics, Korea	African swine fever virus (ASFV) antigen	Ag	Pig	Whole blood	92.5%	100%	[141]
22.	VDRG^®^ FMDV 3Diff/PAN Ag Rapid kit	Median diagnostics, Korea	FMDV serotype specific (O, A and Asia 1) antigen and common antigen detection	Ag	Pig Or Bovine	Saliva, tissue or vesicular fluid	98.35%	100%,	[142]
23.	VETSCAN^®^ Flex4 Rapid Test	Zoetis, USA	*Dirofilaria immitis*	Ag	Canine	Whole blood, serum, or plasma	98.5%	94.0%	[143]
			*Borrelia burgdorferi*	Ab			100%	100%	
			*Ehrlichia canis, E. chaffeensis, and E. ewingii*	Ab			97.4%	97.5%	
			A. *phagocytophilum, A. platys*	Ab			93.3%	96.4%	
24.	WITNESS^®^ Lepto Rapid Test	Zoetis, USA	Leptospires immunoglobulin M (IgM)	Ab	Canine	Serum, or plasma	83.7%	90.2%	[144]
25.	WSSV RP Rapid Test Kit	Innocreate Bioscience, Taiwan	White spot syndrome virus (WSSV)	Ag	Shrimp	Gill tissue	99.21%	100%	[91]

LOD- limit of detection; Ag-Antigen; Ab-Antibody; USA-United States of America; UK- United Kingdom; PFU-Plaque forming unit; Ref. – references.

## 7. Comparative study of LFA with other diagnostic methods

LFAs are platform technologies that play a significant role in diagnostic testing, especially at the point-of-care, due to their inherent merits. They, however, also have limitations compared to other diagnostic methods such as PCR and ELISA (Table 3).

**Table 3.** A comparative analysis of LFAs with two standard alternative diagnostic methods, ELISA and PCR.

Feature	LRA	ELISA	PCR
Speed	Rapid (min) [145]	Moderate (a couple of hours, can be automated) [146]	Slower (hours to days, due to lab processing) [145, 147]
Cost	Low [70]	Moderate to High (requires specialized equipment) [148]	High [147]
Complexity	Simple, easy-to-use [70]	Moderate, requires trained personnel and lab equipment [148]	Complex, requires trained personnel and specialized labs [145, 147]
Portability	High (point-of-care) [70]	Low (typically lab-based) [148]	Low (typically lab-based) [147]
Sensitivity	Generally lower than PCR [145]	High, often semi-quantitative [148]	High (considered the gold standard for many molecular detections) [147]
Specificity	High [147]	High [149]	High [150]
Sample Handling	Easy (various sample types) [70]	Requires venipuncture blood, trained personnel [148]	Requires careful sample collection and transport [147]
Signal Detection	Visual, sometimes with readers [151]	Enzyme-catalyzed colorimetric or fluorescent signal [152]	Amplified molecular signal
Quantification	Qualitative/Semi-quantitative [151]	Often semi-quantitative or quantitative [148]	Quantitative
Key Advantage	Rapid, accessible, cost-effective POC testing	Robust, high-throughput, suitable for antibody/antigen detection	Highly sensitive and specific molecular detection
Key Limitation	Lower sensitivity, potential for false results [153]	Slower than LFA, requires a laboratory setting, can have gray zones [146]	Costly, time-consuming, involves infrastructure [145, 147]

## 8. Regulatory authorities of LFA

Regulation and quality of veterinary LFAs vary widely around the world, and in some areas, no specific regulation exists, which can make it challenging to ensure that the technology remains safe, effective, and a good value for money internationally.

### 8.1. Regulatory networks and quality control measures

Veterinary LFAs used for POCT are classified as medical or in vitro diagnostic devices, and regulations are intended to ensure their safety and effectiveness [45]. However, the quality control of veterinary POCTs is often less rigorous than for human diagnostics. In fact, while medical devices sold in EU countries require CE marking through a conformity assessment process, this requirement is not universally applied to veterinary devices [45]. International organizations have, however, stepped in to address this through using the World Health Organization’s ASSURED criteria [154], which was initially developed for humans but has been modified and applied to veterinary disease diagnostics to drive development where local frameworks are constrained. The WOAH also endorses these initiatives, but legislative power lies in the hands of individual national veterinary associations [154]. A harmonized global approach with similar performance standards for the evaluation of veterinary POCT, taking into account that they are frequently used by users of various skill levels in the field, would be beneficial to both manufacturers and veterinary authorities for assuring diagnostic quality [154].

### 8.2. European Union

In the European Union, the regulation of *in vitro* medical devices (IVDs) intended for POCT differs substantially from human IVDs. By 2020, there were no specific EU regulations for veterinary POCTs of the kind existing in Japan [120]. However, the European Medicines Agency provides oversight of some veterinary-medical aspects, and the European Directorate for the Quality of Medicines and Healthcare works with EMA and the WHO to protect public health by overseeing the quality of veterinary immunological products [155]. While they are not a legal requirement for veterinary diagnostics, the European Union principles described in the *In Vitro* Diagnostic Medical Devices Directive (98/79/EC) for human diagnostics represent reference standards that are frequently applied to validate assays used in veterinary diagnostic applications [156].

### 8.3. Other key markets and emerging regions

The regulatory landscape for veterinary drugs varies significantly across countries and within organizations such as the USA, UK, Japan, Australia, and India [157]. In contrast to Europe, Japan is known for its strict regulation of veterinary POCT products; these are more closely aligned to human in vitro diagnostic standards there than just about anywhere else [120]. In contrast, low- and middle-income countries in the developing world (such as Sub-Saharan Africa and some areas of South Asia) often face substantial regulatory impediments, including time-consuming approval processes that lack transparency and for which most reliable diagnostic tools are unavailable [158]. Some solutions being developed to bridge these gaps include approaches for harmonization to provide common quality and registration standards, strategies to strengthen manufacturing oversight, and post-market surveillance networks. Other challenges include the lack of field-level diagnostics, biosecurity concerns during sampling, and deficient laboratory infrastructure [114]. Regardless, point-of-care tests like the LFAs offer an important and useful approach in advancing disease surveillance and diagnosis, specifically in LMICs, owing to their cost-effectiveness, rapid result generation, and potential for use in resource-limited settings [21].

### 8.4. Quality control standards

Quality assurance and quality control (QA/QC) are essential to support that, whether a regulatory framework exists or not. Those that do exist are, in general, not intrusive and have little or no connection with QA processes and are mainly based on the minimal or reactive approach because of the limited local knowledge of the quality management system [159]. Professional guidelines, including those from the American Society for Veterinary Clinical Pathology, highlight a need for all stages of testing to be taken into account in QA plans and the necessity to verify analyzer function by calibrated activities [160]. They are essential to obtain a correct and fast diagnosis as a basis for an effective treatment [159]. For veterinary LFA, core quality control measures comprise evaluating performance under field or real conditions to check on sensitivity and specificity; providing adequate user training of the test for proper execution and interpretation; and relying on positive and negative controls so as to confirm reliability [154].

## 9. User instruction and LFA implementation

LFAs represent a fast, easy-to-use technique for veterinary diagnostics, especially in decentralized practice. Nevertheless, their broad application and successful integration into the veterinary diagnostic pipeline are hampered by obstacles and require user education.

### 9.1. Obstacles to the implementation of LFAs

Despite the clear advantages that LFAs can offer for veterinary diagnostics, their use in farm animal production is as yet not adequately exploited [161]. A primary obstacle is poor cost-effectiveness, as cattle are commonly held in smallholder operations with little margin for profit, and veterinary tools must be accessible and easy to use [162]. Low validation rates, as well as the absence of regulatory oversight, also impede broad application, with most assays evaluated under laboratory rather than field conditions [163, 154]. Sensitivity limitations also hinder their application, as regular LFAs cannot detect low levels of the analyte or subclinical infection, requiring more advanced biomarker-based models [164]. In addition, LFA interpretation is often supplemented by laboratory testing due to the potential lack of specificity and accuracy [162]. Logistical barriers, such as supply chain limitations, packaging, storage, and distribution constraints, continue to hamper the availability of tests and reagents at users’ sites in the field [154].

### 9.2. Training requirements for LFAs

The success of LFAs for veterinary use relies on practical training, which should address both related technical and interpretive skills. One reason may be the lack of training in quality assurance and quality control (QA/QC) in veterinary laboratories, as this is generally not a priority for suppliers; it involves costs and resources with little impact on production goals [165]. Standardized education and harmonized diagnostic strategies are essential for maintaining accuracy and consistency in human [166] and veterinary clinical microbiology. Diagnostic savvy, disease prevention, and epidemiological skills, as well as the ability to accurately interpret test results, are key competencies for practitioners [167]. Despite being developed for ease of use and rapid field application; adequate user training is still crucial to guarantee reliable results of LFAs [114]. Furthermore, enhancing the accessibility of educational materials and product resources may help bridge the current skills gap, while field epidemiology toolkits provide structured methodologies to help identify and address specific training deficiencies among veterinarians [168].

### 9.3. LFA detection, among other veterinary diagnostic techniques

LFAs are increasingly incorporated into veterinary diagnostic algorithms, and the tools offer convenient and spot-on (pun intended for LFAs) disease identification in an era of real-time decision-making, particularly in resource-challenged settings [163]. The on-site, decentralized nature of the technology enables rapid, low-cost pathogen detection when central laboratory access is limited, especially in rural and low-to-middle-income areas [161]. Submitted LFAs provide rapid turnaround times but typically serve as an initial screening test that necessitates lab-based follow-up because of inferior sensitivity and specificity [162]. Aside from diagnosis, LFAs contribute to surveillance and control of diseases for NIFs by facilitating early detection in the field, as with rabies surveillance programs [169]. They are useful for herd-level health monitoring in a high-throughput manner, as antibody or biomarker detection can be performed rapidly with a low sample amount [170]. Furthermore, ongoing technological progress, such as wearable technologies (such as portable devices) and thermally stable reagents, together with multiplex testing, has improved their performance and reduced costs, making them increasingly suitable for precision livestock health management [154].

## 10. Strategies for ensuring LFA’s stability and performance

To balance performance and consistency in the field for veterinary LFAs, harmonized selection criteria for materials, reagent preservation protocols, device layout, and packaging are required. Bioreceptors must be shielded from heat and moisture by cool, dry storage, desiccation, and stabilizers (e.g., sugars, BSA, agar, or gelatin), with DNA aptamers generally more temperature-resistant than proteins [45]. Stable detection labels dictate that enzyme or dye labels typically rely on maintaining cold chains. However, long-term dry storage at elevated temperatures is possible with protein-enzyme conjugates and nucleic acid enzymes with the aid of protective excipients such as trehalose, polyethylene glycol, and dextran [45].

Nitrocellulose membranes need to be stored in a dry, dark environment with desiccants and have limited shelf life, while their flow/binding properties can drift when exposed to heat/moisture, which has also motivated designs exploring other materials, such as cellulose [171]. Colorimetric reactions, enzymes, and coenzymes could be dry-stored on paper or a chip, and then passively reconstituted during the assay, allowing for a label-free sample to answer operation without cooling down [172]. As antibody stability drops precipitously with increasing temperature and humidity, the manufacturer must establish and demonstrate storage ranges, consider heat spikes during transit, and provide portable temperature and humidity control where indicated, along with moisture-light protection packaging that is not to be frozen [173].

It also relies on systematic optimization of pads, membranes, buffers, surfactants, and geometries (including through dynamic light scattering, optical biosensing, flow modeling, and mechanistic models), with testing over ranges of temperature(s), salt concentrations, and pH values [174]. Finally, robust POC devices should be designed for variable field environments using design frameworks based on MIL STD 810H, reduce user steps with integrated capillary cassettes, and have multilayer active surfaces to offer an extended shelf life of up to six months at room temperature in low-resource settings [175].

## 11. Economic analysis comparing LFAs with other diagnostic approaches

LFAs are an excellent choice for point-of-care testing (POCT) because they are quick and cost-effective [176]. The economic analysis comparing LFAs with other diagnostic approaches is presented in Table 4.

**Table 4.** A comparison of LFAs to culture-based techniques, ELISA, and PCR in veterinary settings based on economic analyses.

Economic Aspect	LFA	Culture-Based Techniques	ELISA	PCR
Test Cost	Low-cost (under $0.50/test); mass production reduces expenses further [170, 177].	Modest direct costs, but owner finances often limit use; part of lab services [178].	Varies: paper-based ~£0.13/sample vs. standard ~£1 [179]; imported kits expensive, recombinant antigens low-cost.	High: £278–£568 per positive result, depending on type; early detection is costly [180].
Operational Costs & Infrastructure	Affordable, operable by untrained staff; suits decentralized, resource-limited settings; minimal infrastructure needed [177].	Requires centralized labs, trained personnel, specific media; hazardous waste disposal adds costs [161].	Needs lab infrastructure and trained staff (less than PCR); antibody prep is expensive, but commercial kits simplify use [181].	Demands specialized labs, molecular biology experts, sophisticated equipment; centralized and expensive [161].
Time to Result	Rapid (5–15 min); enables immediate decisions [170].	Time-consuming (24+ h for incubation, plus 24–48 for susceptibility testing); delays results [182].	Traditional: days; advanced/point-of-care: 15 min; high-throughput fast (24–48 hours turnaround) [148].	24–48 h; time-consuming and may not match disease timing [180].
Accessibility & Portability	Highly portable for field/remote use; no special equipment/electricity; point-of-care testing [170].	Samples must go to central labs; limits rural/immediate access [161].	Generally, lab-based; emerging point-of-care formats; samples are often transported [181].	Low portability; requires lab transport and processing [161].
Overall Cost-Effectiveness & Impact	Highly effective for early detection, preventing losses; disrupts traditional surveillance in low-income areas [183].	Delays cause economic losses (disease spread, mortality); key for targeted therapy but contribute to AMR via empirical treatment [182].	High sensitivity/specificity for some uses; risks of cross-reactivity/false results; commercial kits aid efficiency [184].	Provides detailed info; cost-effective for culture-negative cases; helps prevent AMR costs but is limited by high expense/complexity [180].

## 12. Recent advances and future applications of LFA

### 12.1. Combining molecular techniques

Molecular techniques analyze genetic material, including DNA and RNA. These methods include procedures like RPA, isothermal amplification (akin to LAMP), and PCR. These techniques enable the identification of minute amounts of specific genetic material when coupled with creative technologies such as LFA strips and CRISpen (Clustered Regularly Interspaced Short Palindrome Repeats).

Zhuang et al. [185] combined PCR with LFIA to quantitatively detect canine parvovirus type 2 (CPV-2) by targeting the VP2 gene. They achieved an analytical sensitivity of 3 × 10^1^ copies/μl, demonstrating 100% diagnostic concordance between PCR-LFIA and conventional PCR. Najomtien et al. [186] also provided another PCR-LFD test showing a 50 fg of bacterial gDNA or 1.0 CFU detection limit for *Burkholderia pseudomallei*.

By using LAMP in conjunction with LFA, many targets can be detected simultaneously in a single test, thereby improving analytical efficiency and economy. Typically, biotin and fluorescein isothiocyanate (FITC), either DIG or biotin, and the primers used in LAMP-LFA are made with two haptens at their ends. While the other hapten (FITC or DIG) connects to matching antibodies conjugated to gold nanoparticles, biotin binds to its counterpart, such as streptavidin, which is immobilized on the LFA strip [27].

Jang et al. [187] constructed an influenza A/B multiplex LAMP-LFA and tested it on clinical samples, finding it to be 94.1% sensitive for influenza A and 96.6% sensitive for influenza B. It was also 98% specific for uninfected samples. The findings demonstrate that the influenza A/B multiplex LAMP-LFA is dependable for use in resource-limited settings. Additionally, LAMP combined with nanoparticle-based lateral flow biosensors targeting the ATI gene of MPXV allows for the detection of MPXV strains without cross-reacting with non-MPXV pathogens [188]. Another excellent example of this is the combination of isothermal amplification and lateral flow testing for detecting bovine coronavirus, which shows that it is just as selective and sensitive as RT-qPCR approaches [189].

RPA is a method of isothermal amplification that uses single-stranded binding proteins, DNA polymerases, and recombinase enzymes. The resultant amplicon can be identified with a lateral flow test that incorporates anti-FAM gold conjugates and biotin-ligand molecules for direct visual interpretation [190]. Onchan et al. [191] demonstrated the efficacy of a lateral flow dipstick (RPA-LFD) in recombinase polymerase amplification for detecting Babesia in dogs, achieving a minimal detection limit of 22.5 copies/μl (0.1 fg/µl).

CRISPR/Cas, which stands for clustered regularly interspaced short palindromic repeats, represents a major advance in genome editing. It works well for finding nucleic acids for diagnosis [42]. Osborn et al. [192] developed an LFA using CRISPR/Cas9 technology to find SARS-CoV-2 sequences with single-base specificity. To rapidly identify and differentiate four viral respiratory pathogens, like influenza A and B, respiratory syncytial virus, and SARS-CoV-2, in a single assay, the research team developed a multiplex fluorescence test. Additionally, a study by Huyen et al. [193] demonstrated the application of a lateral-flow readout using CRISPR-Cas cleavage of LAMP amplicons to diagnose *N*. *meningitidis*. These findings illustrate the potential application of CRISPR/Cas9 technology in point-of-care diagnostics.

LFA strips incorporated with surface-enhanced Raman scattering (SERS) are very sensitive for disease diagnosis and capable of detecting numerous targets at once. The test and control lines change color when particular biomarkers are present. These strips use Raman reporter-labeled nanotags to find these biomarkers. The Raman peak intensity of the reporter molecule makes it possible to accurately measure the target analytes. Wang et al. [194] produced a SERS-based LFA strip that was very sensitive in less than 15 min for finding *Bacillus anthracis, Francisella tularensis*, and *Yersinia pestis* using a 40 μl pathogen sample. Early infectious illness detection in field settings is made possible by the downsizing of Raman equipment and SERS-based LFAs.

### 12.2. Advancement in labeling probes

Proper labeling probes, functionalization methods, and conjugation processes are crucial for enhancing the sensitivity and specificity of LFIAs for detecting various target molecules. In addition to gold nanoparticles, recent advancements have introduced a range of labeling probes, including latex particles, magnetic nanoparticles, quantum dots, and more recently, carbon nanoparticles, silica nanoparticles, europium nanoparticles, and upconversion nanoparticles [54].

Recent developments in quantum dot lateral flow immunoassay strips have presented them as an ideal alternative to traditional luminescent dyes. These fluorescent labels exhibit unique optoelectronic properties, including high molecular extinction coefficients and remarkable stability against photochemical decomposition, making them suitable for detecting various analytes, such as tumors, biomolecules, bacteria, and viruses [195]. Luminescent, water-soluble, carboxyl-functionalized quantum dots conjugated with streptococcal protein G have been used in QD-based lateral flow immunoassays to identify anti-PPRV antibodies. When recognizing PPR serum IgG antibodies, this method provides more sensitivity than competitive enzyme-linked immunosorbent assays and immunochromatographic lateral flow devices [196].

Using magnetic nanoparticles (MNPs) as detecting labels, which are sensed and quantified by a magnetic signal reader, facilitates the development of immunochromatographic tests with quantitative capabilities [197]. They can also be employed in immunomagnetic separation, enhancing selectivity and sensitivity. The combination of the superparamagnetic properties of Fe_3_O_4_ and the surface chemistry of gold (Au) made Fe_3_O_4_/Au core/shell nanoparticles, which are easy to separate and functionalize, resulting in improved sensitivity. The magnetic LFAs are effective for detecting and quantifying pathogens, biomarkers, and small molecules such as toxins, allergens, and drugs [198].

### 12.3. Optimizing LFA design and readout systems

Advancements in labeling probes require integrating LFAs with innovative readout systems. Modifications in device design and optimization of assay parameters enhanced the precision, detection limits, and reliability of LFAs. The optimization results transition from qualitative interpretations to semi-quantitative assessments. Integration of readout systems, such as magnetic sensors [198], UCNP readers [33], SERS readers [43, 199], photoacoustic readers [200], thermal contrast readers [201], and fluorescence resonance energy transfer readers [202], enhances LFA’s sensitivity and accuracy. The integration minimizes complexity and the potential for false positives. The advancement of smartphone technologies offers a significant opportunity for improving LFA results interpretation through high-resolution cameras, computational power, and networking capabilities [203, 204].

Additionally, integration of lateral flow with artificial intelligence is crucial for point-of-care immunoassays, enabling the collection, analysis, and interpretation of real-time data [205, 206]. The integration with digital platforms and improved multiplexing capabilities will enhance the authenticity of LFAs, ensuring greater accuracy and faster, more intelligent detection, ultimately leading to better outcomes.

### 12.4. Robotic-based LFA application

The integration of robotics represents a significant advancement in diagnostic fields. Anderson et al. [207] presented an automated robotic liquid handling system tailored for the LFA development process. This system effectively carries out a variety of assay development experiments with both discrete and continuous variables. It reduces hands-on time while increasing study size. The researchers successfully identified the most effective monoclonal antibodies (MAbs) for the swift and consistent creation of LFAs for malaria and M. tuberculosis, thereby reducing costs and expediting the LFA development process.

## 13. Conclusions

LFAs are very important for diagnosing and keeping updated on infectious diseases in point-of-care settings, especially in resource-limited areas. Because they are easy to use, cheap, and quick to understand, LFAs are very important in veterinary industries like aquaculture, livestock, and pets. LFAs can find a lot of different pathogens, such as viral and bacterial infections and parasitic disorders, as well as cross-border diseases of animals, like FMD, AIV, rabies, and RVF. Recent improvements in LFA technology, including multiplexing, combining with molecular approaches, and new labeling probes, have made it much easier to diagnose diseases. Nonetheless, LFAs encounter constraints, including qualitative result interpretation and the necessity for confirmation diagnosis. However, continuous improvement in the design optimization, readout systems, artificial intelligence, and robotic automation promises to overcome these challenges with improved accuracy and reproducibility. LFAs will play a vital role in sustainable agriculture and public health by integrating with cutting-edge technologies, expanding into new diagnostic applications, and broader adoption in global health initiatives.

## Data Availability

The data presented in this study are available from the corresponding author upon reasonable request.

## List of abbreviations

LFAs, Lateral flow assays; FMD, foot-and-mouth disease; HS, hemorrhagic septicemia; PPR, peste des petits ruminant; LSD, lumpy skin disease; LMICs, low and middle-income countries; PCR, polymerase chain reaction; NGS, next-generation sequencing; MALDI-TOF MS, matrix-assisted laser desorption/ionization-time-of-flight mass spectrometry; WOAH, World Organization for Animal Health; POC, point-of-care; LAMP, loop-mediated isothermal amplification; RPA, biosensors and recombinase polymerase amplification; ELISA, enzyme linked immune-sorbent assay; hCG, human chorionic gonadotropin; HIV, human immunodeficiency virus; CRISPR, clustered regularly interspaced short palindromic repeats; CV, constant of variance; FAO, Food and Agriculture Organization; TADs, Transboundary Animal Diseases; SARS, severe acute respiratory syndrome; MERS, Middle East respiratory syndrome; AIVs, avian influenza viruses; HPAI, highly pathogenic avian influenza; LFIA, lateral flow immunochromatographic assay; CyHV-3, Cyprinid herpesvirus 3; KHVD, koi herpesvirus disease; WSSV, White Spot Syndrome Virus; LD, limit of detection; CFU, colony-forming units, AHPND, acute hepatopancreatic necrosis disease; LFD, lateral flow device; mAbs, monoclonal antibodies; GNP-LFT, gold nanoparticle lateral flow test; dRIT, direct rapid immunohistochemistry test, DFA, direct fluorescent antibody, RT-PCR, reverse-transcription polymerase chain reaction; FIV, feline immunodeficiency virus, FPV, feline panleukopenia virus, CPV, canine parvovirus canine CPV-2, parvovirus type 2; FITC, fluorescein isothiocyanate; LFBs, lateral flow biosensors; SERS, surface-enhanced Raman scattering.
